# *Staphylococcus aureus* Ocular Infection: Methicillin-Resistance, Clinical Features, and Antibiotic Susceptibilities

**DOI:** 10.1371/journal.pone.0042437

**Published:** 2012-08-07

**Authors:** Chih-Chun Chuang, Ching-Hsi Hsiao, Hsin-Yuan Tan, David Hui-Kang Ma, Ken-Kuo Lin, Chee-Jen Chang, Yhu-Chering Huang

**Affiliations:** 1 Department of Ophthalmology, Chang Gung Memorial Hospital, Linkou, Taiwan; 2 Department of Ophthalmology, Changhua Christian Hospital, Changhua, Taiwan; 3 Department of Ophthalmology, Yuan-Sheng Hospital, Changhua, Taiwan; 4 College of Medicine, Chang Gung University, Taoyuan, Taiwan; 5 Graduate Institute of Clinical Medical Science, Chang Gung University, Taoyuan, Taiwan; 6 Clinical Informatics and Medical Statistics Research Center, Chang Gung University, Taoyuan, Taiwan; 7 Division of Pediatric Infectious Diseases, Department of Pediatrics, Chang Gung Memorial Hospital, Linkou, Taiwan; University of Birmingham, United Kingdom

## Abstract

**Background:**

Methicillin-resistant *Staphylococcus aureus* (MRSA) infection is an important public health issue. The study aimed to determine the prevalence of ocular infections caused by MRSA and to identify the clinical characteristics and antibiotic susceptibility of ocular MRSA infections by comparing those of ocular methicillin-sensitive *S. aureus* (MSSA) infections.

**Methodology/Principal Findings:**

The medical records of the patients (n = 519) with culture-proven *S. aureus* ocular infections seen between January 1, 1999 and December 31, 2008 in Chang Gung Memorial Hospital were retrospectively reviewed. Two hundred and seventy-four patients with MRSA and 245 with MSSA ocular infections were identified. The average rate of MRSA in *S. aureus* infections was 52.8% and the trend was stable over the ten years (*P* value for trend  = 0.228). MRSA ocular infections were significantly more common among the patients with healthcare exposure (*P* = 0.024), but 66.1% (181/274) patients with MRSA ocular infections had no healthcare exposure. The most common clinical presentation for both MRSA and MSSA ocular infections was keratitis; MRSA and MSSA caused a similar disease spectrum except for lid infections. MRSA was significantly more resistant than MSSA to clindamycin, erythromycin and sulfamethoxazole/trimethoprim (all *P*<0.001).

**Conclusions/significance:**

We demonstrated a paralleled trend of ocular MRSA infection in a highly prevalent MRSA country by hospital-based survey. Except for lid disorder, MRSA shared similar spectrum of ocular pathology with MSSA. Since *S. aureus* is a common ocular pathogen, our results raise clinician’s attention to the existence of highly prevalent MRSA.

## Introduction

*Staphylococcus aureus* is among the most important and commonly isolated human bacterial pathogens. *S. aureus* isolates resistant to methicillin, usually also resistant to other β-lactam antimicrobial drugs, are termed methicillin-resistant *S. aureus* (MRSA). MRSA, first identified in the 1960s, was traditionally associated with healthcare facilities, but is now a dominant pathogen in community-associated infections. [1] MRSA is of particular concern as a serious cause of morbidity and mortality worldwide, because of its multiple drug resistance, leaving limited treatment options and its believed increasing prevalence.

Previous data on ocular MRSA infections were generally limited to case reports and small case series, [2]–[7] but a variety of more recent publications have presented substantially larger analyses of ocular MRSA in the United States. [8]–[18] Case series of catastrophic eye infections caused by MRSA has been reported recently in patients after refractive and cataract surgery. [11], [15], [16] According to the report from American Society of Cataract and Refractive Surgery, MRSA has replaced nontuberculous mycobacteria to be the most common pathogen causing infections after laser-assisted in situ keratomileusis. [19] MRSA has been reported to account for 18.2% (6/33) culture-proven endophthalmitis in a referral vitreoretinal practice. [15] The proportion of MRSA in ocular *S. aureus* infections from a single institution varies from 3% to 30%, with some reports showing increasing incidence of MRSA. [8]–[10] The Surveillance Network, which monitors antimicrobial susceptibility patterns of bacterial pathogens in the United States, reported an increase in the proportion of MRSA among *S. aureus* ocular infections, from 29.5% in 2000 to 41.6% in 2005, which showed MRSA is a rising menace in ocular field. [12] In Taiwan, the rate of MRSA among *S. aureus* clinical isolates, was about 60% during 1997–2000, [20], [21] a rate higher than that reported in other regions of the world, [20] but the rate of ocular infections caused by MRSA remains unknown.

Here, we conducted a 10-year retrospective study to determine the rate of ocular MRSA infections and to identify the clinical characteristics and antibiotic susceptibility of ocular MRSA infection by comparing those of ocular methicillin sensitive *S. aureus* (MSSA) infections seen in Chang Gung Memorial Hospital, a 3000-bed tertiary referral hospital in Taiwan.

## Methods

### Ethics

The study was approved by the institute review boards from Chang Gung Memorial Hospital, which allowed retrieve of the patients list from the electronic microbiology database, review of the medical information. A waiver of consent was granted given the retrospective nature of the project and anonymous analysis of the data.

### Participants and Procedures

From the microbiologic laboratory database, we identified all the patients with an ocular specimen, collected by ophthalmologists, sent for bacterial culture and positive for *S. aureus* between January 1, 1999 and December 31, 2008. We included no more than one isolate per patient. We determined susceptibility of the isolates to seven antibiotics (oxacillin, penicillin, erythromycin, clindamycin, trimethoprim/sulfamethoxazole, vancomycin and teicoplanin) using the disc diffusion method according to the Clinical and Laboratory Standard Institute (CLSI) standards for antimicrobial susceptibility testing. We used oxacillin, which was replaced by cefoxitin since March 2006, to test for β**-**lactam antibiotic resistance. We reviewed patient charts to collect demographic and clinical information. Based on the structures involved, we classed ocular infections into one of seven diagnoses: conjunctivitis, keratitis, lid disorder, lacrimal system disorder, wound infection, endophthalmitis and other (e.g., blebitis, buckle or implant infection and sclera ulcer). If the chart showed more than one diagnosis, we chose the primary pathology or the more severe diagnosis. If the patients had either: 1) a MRSA infection identified after 48 hours of admission to a hospital; 2) a history of hospitalization, surgery, dialysis, or residence in a long-term care facility within one year of the MRSA culture date; 3) a permanent indwelling catheter or percutaneous medical device present at the time of culture; or 4) a known positive culture for MRSA prior to the study period, they were thought to have healthcare exposure [22].

### Statistical Analysis

Patients with MSSA ocular infections constituted the control group and patients with MRSA ocular infections were the study group. Nominal variables were analyzed with the chi square test. Continuous variables were analyzed with Student’s *t* test. Trend analysis was performed by the chi square test for trends. For comparison, we grouped the data into two five-year study periods, from January 1999 to December 2003 and from January 2004 to December 2008. All analyses were two-tailed, and *P*<0.05 was considered statistically significant. We performed all statistical analyses using R (The R Foundation for Statistical Computing, Vienna, Austria; available at: http://www.R-project.org).

## Results

During the 10-year study period, *S. aureus* was isolated from 519 patients. Of these, 274 were MRSA and 245 were MSSA.

### The Rate of MRSA in *S. aureus* Ocular Infections

**Figure 1:**
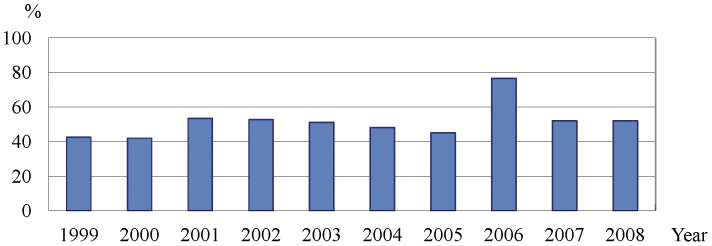
Percentage of patients with ocular methicillin-resistant *Staphylococcus aureus* (MRSA) by year.

As illustrated in Figure 1, the average annual rate of MRSA among ocular *S. aureus* infections was 52.8%, ranging from 41.9% in 2000 to 76.5% in 2006, and the trend was stable for the 10-year interval (*P* value for trend  = 0.228).

### Characteristics of Ocular MRSA and MSSA Infections (Table 1)

The groups had similar median ages, 41.3 years (range, 1 month to 89 years) for patients with ocular MRSA infections and 37.3 years (range, 2 months to 84 years) for those with ocular MSSA infections. Of the 281 males and 238 females infected, significantly more males than females had MSSA (*P* = 0.036). The groups did not differ in eye involvement (laterality). Ninety three patients (93/274, 33.9%) with MRSA ocular infections and 60 patients (60/245, 24.5%) with MSSA ocular infections had healthcare exposure; MRSA infections were significant more common among the patients with healthcare exposure (*P* = 0.024). Patients with MRSA did not differ significantly from MSSA patients in the presence of underlying comorbidities or use of immunosuppressants. In MRSA group, the rate of the patients with healthcare exposure significantly decreased from 44.8% to 25.9% (*P* = 0.002) in the most recent five years. There was no change in other demographics of both MRSA and MSSA groups between the two study periods.

**Table 1:** Comparison of demographics and characteristics of ocular 274 methicillin-resistant *Staphylococcus aureus* and 245 methicillin-sensitive *Staphylococcus aureus* infections.

Characteristics	MRSA (n = 274)	MSSA (n = 245)	*p* value*
**Age** (Mean ±SD years)	41.3±26.6	37.3±27.3	0.096
**Gender** (F/M)	138/136	100/145	0.036
**Eye** (R/L/B)	123/115/36	106/110/29	0.776
**Healthcare exposure**	93	60	0.024
**Comorbidities**	82	57	0.218
**Immunosuppressive treatment** (Local/Systemic/Nil)	17/20/237	11/8/226	0.079

* Two-sample *t* test for age comparison, Chi-square test for others.

MRSA: methicillin-resistant *Staphylococcus aureus*, MSSA: methicillin-sensitive *Staphylococcus aureus*, F: female, M: male, R: right eye, L: left eye, B: both eyes.

### Clinical Diagnoses Associated with Ocular MRSA and MSSA Infections (Table 2)

Keratitis was the most common ocular diagnosis in all patients and accounted for 36.1% and 40.0% of cases of MRSA and MSSA, respectively. About 20% of patients in both groups had conjunctivitis. In MRSA group, the rate of lid disorder significantly increased from 23.9% to 76.1% (*P*<0.001), while the rate of keratitis significantly decreased from 55.6% to 44.4% during the last five years (*P* = 0.001). The rate of various diagnoses caused by MSSA did not change between the two study periods. By comparison, patients with MRSA infections presented with lid disorder significantly more often than patients with MSSA infections (24.5% vs. 16.7%, *P* = 0.040), but the rate of patients presenting with other diagnoses did not differ significantly between both groups. MRSA and MSSA did not differ in their association with a diagnosis of vision-threatening disorders (i.e., keratitis, orbital cellulitis or endophthalmitis).

**Table 2:** Comparison of clinical diagnoses associated with ocular 274 methicillin-resistant *Staphylococcus aureus* and 245 methicillin-sensitive *Staphylococcus aureus* infections.

Diagnosis	MRSA (n = 274)No. (%)	MSSA (n = 245)No. (%)	*p*-value
**Keratitis**	99 (36.1)	98 (40.0)	0.414
**Lid disorder** *	67 (24.5)	41 (16.7)	0.040
**Conjunctivitis**	55 (20.1)	49 (20.0)	1
**Lacrimal system disorder** †	29 (10.6)	37 (15.1)	0.158
**Wound infection**	10 (3.6)	7 (2.9)	0.795
**Endophthalmitis**	9 (3.3)	4 (1.6)	0.357
**Others** ‡	5 (1.8)	9 (3.7)	0.305
**Vision-threatening disorder** §	131 (47.8)	121 (49.4)	0.786

* Lid disorders including cellulitis, lid abscess and hordeolum.

† Lacrimal system disoder including dacrocystitis and canaliculitis.

‡ Others including blebitis, sclera ulcer, buckle infection and hydroxyapatite implant infection.

§ Vision-threatening disorder including keratitis, cellulitis and endophthalmitis.

MRSA: methicillin-resistant *Staphylococcus aureus*, MSSA: methicillin-sensitive *Staphylococcus aureus.*

### Antibiotics Susceptibility of MRSA and MSSA for Ocular Infections (Table 3)

As expected, MRSA was significantly more resistant than MSSA to several antibiotics including clindamycin, erythromycin and sulfamethoxazole/trimethoprim. Seventy-eight percent of MRSA isolates were susceptible to sulfamethoxazole/trimethoprim. All MRSA and MSSA isolates were susceptible to vancomycin and teicoplanin. Susceptibility of both MRSA and MSSA groups to all seven antibiotics did not change significantly in the most recent five years.

**Table 3:** Antibiotic susceptibility of 274 methicillin-resistant *Staphylococcus aureus* and 245 methicillin-sensitive *Staphylococcus aureus* isolates for ocular infections.

Antibiotics	MRSA (n = 274)No. (%)	MSSA (n = 245)No. (%)	*p* value†
**Clindamycin**	24 (8.8)	179 (73.1)	<0.001
**Erythromycin**	14 (5.1)	153 (62.5)	<0.001
**Penicillin**	0 (0)	20 (8.2)	<0.001
**Sulfamethoxazole/Trimethoprim**	214 (78.1)	241 (99.2)	<0.001
**Vancomycin**	274 (100)	245 (100)	
**Teicoplanin** *	260 (100)	226 (100)	

* No sensitivity test for teicoplanin in 1999.

† Two-proportional *t* test.

MRSA: methicillin-resistant *Staphylococcus aureus*, MSSA: methicillin-sensitive *Staphylococcus aureus.*

## Discussion

To our knowledge, the current study represents the largest reported case series of ocular *S. aureus* infections. Our findings show that 52.8% of ocular *S. aureus* infections were MRSA and the trend was stable over a 10-year interval at our hospital. MRSA ocular infections were significant more common among the patients with healthcare exposure. Except for lid disorder, MRSA and MSSA caused a similar disease spectrum and severity of ocular disorders. MRSA was significantly more resistant than MSSA to clindamycin, erythromycin and sulfamethoxazole/trimethoprim.

Our data showed a relatively high and stable rate of ocular isolates of MRSA in our hospital, although most other studies reported increasing MRSA prevalence. Blomquist showed an increase in the incidence of ophthalmic MRSA from 12% to 33% over a 5-year period (2000 to 2004) in an urban health care system in the United States. [9] Freidlin et al. reported a similar increase in the proportion of ocular MRSA infections from 4.1% in 1998 to 1999 to 16.7% in 2005–2006. [10] The Surveillance Network demonstrated an increase in the proportion of culture-positive ocular MRSA from 29.5% in 2000 to 41.6% in 2005 in serious *S. aureus* ocular infections, predicting that MRSA could be more common than MSSA within two to three years, based on the rate of increase. [13] Instead, our results were in line with the prevalence reported for our hospital and Taiwan. MRSA was first documented in the early 1980s in Taiwan and its prevalence has increased remarkably since. [23] Based on data from 12 major hospitals in Taiwan, MRSA accounted for 53% to 83% of all *S. aureus* clinical isolates in 2000. [24] In our hospital, an average of 63.9% of all *S. aureus* infections were MRSA (range, 59.0% to 70.0%), with no trend change over a 10-year interval, similar to ocular infections due to *S. aureus*. These data indicate that MRSA is prevalent and with a stable rate of *S. aureus* infections in our hospital, perhaps in the plateau stage, in the past decade.

As expected, a statistically significantly greater number of the patients with MRSA ocular infections had healthcare exposure than those with MSSA ocular infections in this study. However, it is noteworthy that two thirds of the patients (181/274, 66.1%) with MRSA ocular infections had no healthcare exposure, which meant the isolates were potentially community associated. MRSA was once associated with healthcare facilities, but more recent reports showed an increasing frequency of isolates from community-associated MRSA infections in Taiwan as elsewhere. [25]–[30] Since most ophthalmologic patients are seen and treated as outpatients instead of inpatients, community-associated MRSA may play an important role in MRSA ocular infections. Further analysis of community associated ocular MRSA infection has been conducted and published separately [31].

In our study, the most common presentation of ocular MRSA infections was keratitis (36.1%), followed by lid disorder (24.5%) and conjunctivitis (20.1%); nearly half (47.8%) of ocular MRSA infections were vision-threatening. However, previous large case series studies showed that the most common manifestation of ophthalmic MRSA infection was conjunctivitis [8], [10] or lid disorder [9]; vision-threatening infections were relatively uncommon. [8]–[10] Our results may have differed due to selection bias, because there were more severe cases in our hospital, a tertiary referred center. Also, physicians may differ in which cases they sent for diagnostic testing; some may tend to culture only the most serious cases. Third, we may exclude some patients with ocular infections, while the cultures were not done by ophthalmologists.

MRSA is believed to cause a more severe disease than MSSA, but this observation has not reached consensus. [32], [33] Our results did not show that MRSA caused more severe ocular diseases than MSSA; this agrees with Freidlin’s study, which reported MRSA and MSSA caused similar eye disease. [10] We did find that patients with MRSA were more likely to have lid infections. In addition, the rate of lid disorder caused by MRSA significantly increased, but the rate of keratitis caused by MRSA significantly decreased during the last five years. Community-associated MRSA has a reported predilection for causing skin and soft tissue infections, [22], [34] and we also found that lid and lacrimal system disorders were more common, but keratitis, endophthalmitis and wound infection were less common among community associated MRSA cases than healthcare associated MRSA cases. [31] Thus, 66.1% of patients with MRSA ocular infections were community-associated and the paralleled significant increase in the rate of the MRSA patients without healthcare exposure (i.e. community-associated MRSA) in the most recent five years may explain these results.

According to antibiotic susceptibility profiles, vancomycin was the most active agent against ocular MRSA isolates, whereas sulfamethoxazole-trimethoprim retained some degree of activity against MRSA, but was less effective than in previous studies. [9], [10], [13] Although vancomycin retains extremely high efficacy against MRSA, *S. aureus* with reduced susceptibility to vancomycin was identified. [35] Since prior vancomycin use is a risk factor for MRSA with reduced vancomycin susceptibility, [36] and no convincing evidence shows that routine vancomycin prophylaxis is effective in elective cataract surgery, [37] we recommend that ophthalmologists follow guideline of the Centers for Disease Control and Prevention [38] and the American Academy of Ophthalmology [39] against the routine use of vancomycin for prophylaxis to halt the spread of resistance. Several recent studies have reported that MRSA has a high rate of *in vitro* resistance to fluoroquinolones, including new generation ones, the most popular empiric therapy in ocular infections. [9], [10], [12], [13], [15], [16], [40] We did not test fluoroquinolones in our study because they were not included in the recommended list of antibiotics published by the CLSI. In Taiwan, National data from 2000 (TSAR program) has demonstrated 40% *S. aureus* (including MSSA and MRSA) in vitro resistance to ciprofloxacin. [21] We may extend the antibiotic susceptibility profiles to include commonly used topical antibiotics in future studies.

Eight ocular MRSA isolates from pediatric patients were stored and available for genotyping analysis, including pulsed-filed gel electrophoresis (PFGE) typing, SCC*mec* elements and the detection of PVL genes in this study. The only one healthcare associated MRSA isolate was characterized as PFGE type A/SCC*mec* IIIA/PVL-negative, which was compatible with those of healthcare associated MRSA isolates (sequence type (ST) 239, Hungary clone) in our previous studies. [29], [41] Four of seven isolates classified as community-associated MRSA were characterized by PFGE type D/SCC*mec* VT/PVL positive, and the other three were characterized by PFGE type C/SCCmec IV/PVL-negative. Both clones shared the common genetic characteristics of community-associated MRSA strains in Taiwan (ST 59, Taiwan clone). [29], [41] These molecular results, though limited, further confirmed the classification of community-associated and healthcare-associated MRSA based on epidemiologic data in the present study was confident.

The currently recommended disc diffusion method to determine resistance against methicillin for *S. aureus* uses cefoxitin rather than oxacillin, because cefoxitin results are easier to interpret and are more sensitive for the detection of *mecA*-mediated resistance than oxacillin results, especially for identifying community associated MRSA, which may have low MIC to oxacillin. [42], [43] Our Clinical Microbiology Laboratories started using cefoxitin instead of oxacillin to test for β**-**lactam antibiotic resistance since March 2006 as the performance standard from the CLSI was revised. When we made a comparison between MRSA and MSSA ocular infections from March 2006 to the end of 2008, the results were the same as those in the 10-year study interval except there was no significant difference in the proportion of the patients with healthcare exposure in MRSA and MSSA. It was probably due to the increase in community-associated MRSA over time in our hospital [31].

Our study has the inherent flaws of a retrospective design. The patient selection criteria may influence data interpretation. Since our study population attended a referral-based, tertiary-care hospital, results may not be applicable to other populations. In addition, we used oxacillin/cefoxitin testing as a surrogate for detecting the *mec*A gene in the identification of resistant species of *Staphylococcus*; and we distinguished community-associated MRSA from healthcare-associated MRSA based on epidemiological differences, not genetic characterization. Our Clinical Microbiology Laboratories retain only isolates from blood for long-term storage, so we did not have the ocular isolates for further analysis. Thus, misclassification bias may limit applicability of the results. Furthermore, resistance found *in vitro* based on serum systemic standards does not always correlate with clinical resistance, because there are no susceptibility standards for topical therapy. Finally, the scope of our study primarily focused on the epidemiology and included a broad spectrum of diseases, so the treatment and visual outcomes were not intended to be discussed.

Infectious diseases may differ by regions in epidemiologic patterns, spectrum and severity of disease, and profiles of antibiotic susceptibility. In this 10-year retrospective study, we found that MRSA was common in ocular *S. aureus* infections in our hospital, which paralleled trends of systemic MRSA infections, in this highly prevalent MRSA country. While we failed to demonstrate a difference in virulence between MRSA and MSSA, vision-threatening disorders were common in both. All MRSA isolates were susceptible to vancomycin. Establishing the baseline characteristics of MRSA ocular infections helps us track future progress and choose the most appropriate treatment.

## References

[1] Deresinski S (2005) Methicillin-resistant Staphylococcus aureus: an evolutionary, epidemiologic, and therapeutic odyssey. Clin Infect Dis 40: 562–573. 15712079 10.1086/427701

[2] Fukuda M, Ohashi H, Matsumoto C, Mishima S, Shimomura Y (2002) Methicillin-resistant Staphylococcus aureus and methicillin-resistant coagulase-negative Staphylococcus ocular surface infection efficacy of chloramphenicol eye drops. Cornea 21: S86–89. 12484705 10.1097/01.ico.0000263125.99262.42

[3] Sotozono C, Inagaki K, Fujita A, Koizumi N, Sano Y, et al (2002) Methicillin-resistant Staphylococcus aureus and methicillin-resistant Staphylococcus epidermidis infections in the cornea. Cornea 21: S94–101. 12484707 10.1097/01.ico.0000263127.84015.3f

[4] Donnenfeld ED, O’Brien TP, Solomon R, Perry HD, Speaker MG, et al (2003) Infectious keratitis after photorefractive keratectomy. Ophthalmology 110: 743–747. 12689896 10.1016/S0161-6420(02)01936-X

[5] Kotlus BS, Rodgers IR, Udell IJ (2005) Dacryocystitis caused by community-onset methicillin-resistant Staphylococcus aureus. Ophthalmic Plastic & Reconstructive Surgery 21: 371–375. 16234703 10.1097/01.iop.0000175035.22953.71

[6] Kotlus BS, Wymbs RA, Vellozzi EM, Udell IJ (2006) In vitro activity of fluoroquinolones, vancomycin, and gentamicin against methicillin-resistant Staphylococcus aureus ocular isolates. American Journal of Ophthalmology 142: 726–729. 17056356 10.1016/j.ajo.2006.06.030

[7] Rutar T, Chambers HF, Crawford JB, Perdreau-Remington F, Zwick OM, et al (2006) Ophthalmic manifestations of infections caused by the USA300 clone of community-associated methicillin-resistant Staphylococcus aureus. Ophthalmology 113: 1455–1462. 16766029 10.1016/j.ophtha.2006.03.031

[8] Shanmuganathan VA, Armstrong M, Buller A, Tullo AB (2005) External ocular infections due to methicillin-resistant Staphylococcus aureus (MRSA). Eye 19: 284–291. 15375372 10.1038/sj.eye.6701465

[9] Blomquist PH (2006) Methicillin-resistant Staphylococcus aureus infections of the eye and orbit (an American Ophthalmological Society thesis). Transactions of the American Ophthalmological Society 104: 322–345. 17471350 PMC1809917

[10] Freidlin J, Acharya N, Lietman TM, Cevallos V, Whitcher JP, et al (2007) Spectrum of eye disease caused by methicillin-resistant Staphylococcus aureus. American Journal of Ophthalmology 144: 313–315. 17659970 10.1016/j.ajo.2007.03.032

[11] Solomon R, Donnenfeld ED, Perry HD, Rubinfeld RS, Ehrenhaus M, et al (2007) Methicillin-resistant Staphylococcus aureus infectious keratitis following refractive surgery. American Journal of Ophthalmology 143: 629–634. 17320811 10.1016/j.ajo.2006.12.029

[12] Asbell PA, Colby KA, Deng S, McDonnell P, Meisler DM, et al (2008) Ocular TRUST: nationwide antimicrobial susceptibility patterns in ocular isolates. Am J Ophthalmol 145: 951–958. 18374299 10.1016/j.ajo.2008.01.025

[13] Asbell PA, Sahm DF, Shaw M, Draghi DC, Brown NP (2008) Increasing prevalence of methicillin resistance in serious ocular infections caused by Staphylococcus aureus in the United States: 2000 to 2005. J Cataract Refract Surg 34: 814–818. 18471638 10.1016/j.jcrs.2008.01.016

[14] Cavuoto K, Zutshi D, Karp CL, Miller D, Feuer W (2008) Update on bacterial conjunctivitis in South Florida. Ophthalmology 115: 51–56. 17572497 10.1016/j.ophtha.2007.03.076

[15] Deramo VA, Lai JC, Winokur J, Luchs J, Udell IJ (2008) Visual outcome and bacterial sensitivity after methicillin-resistant Staphylococcus aureus-associated acute endophthalmitis. Am J Ophthalmol 145: 413–417. 18191097 10.1016/j.ajo.2007.10.020

[16] Major JC Jr, Engelbert M, Flynn HW Jr, Miller D, Smiddy WE, et al. (2010) Staphylococcus aureus endophthalmitis: antibiotic susceptibilities, methicillin resistance, and clinical outcomes. Am J Ophthalmol 149: 278–283 e271. 19926069 10.1016/j.ajo.2009.08.023

[17] Adebayo A, Parikh JG, McCormick SA, Shah MK, Huerto RS, et al (2011) Shifting trends in in vitro antibiotic susceptibilities for common bacterial conjunctival isolates in the last decade at the New York Eye and Ear Infirmary. Graefes Archive for Clinical and Experimental Ophthalmology 249: 111–119. 10.1007/s00417-010-1426-6 20532549

[18] Haas W, Pillar CM, Torres M, Morris TW, Sahm DF (2011) Monitoring Antibiotic Resistance in Ocular Microorganisms: Results From the Antibiotic Resistance Monitoring in Ocular MicRorganisms (ARMOR) 2009 Surveillance Study. Am J Ophthalmol 152: 567–574 e563. 21652021 10.1016/j.ajo.2011.03.010

[19] Solomon R, Donnenfeld ED, Holland EJ, Yoo SH, Daya S, et al (2011) Microbial keratitis trends following refractive surgery: results of the ASCRS infectious keratitis survey and comparisons with prior ASCRS surveys of infectious keratitis following keratorefractive procedures. J Cataract Refract Surg 37: 1343–1350. 21700112 10.1016/j.jcrs.2011.05.006

[20] Diekema DJ, Pfaller MA, Schmitz FJ, Smayevsky J, Bell J, et al (2001) Survey of infections due to Staphylococcus species: frequency of occurrence and antimicrobial susceptibility of isolates collected in the United States, Canada, Latin America, Europe, and the Western Pacific region for the SENTRY Antimicrobial Surveillance Program, 1997–1999. Clin Infect Dis 32 Suppl 2S114–132. 11320452 10.1086/320184

[21] McDonald LC, Lauderdale TL, Shiau YR, Chen PC, Lai JF, et al (2004) The status of antimicrobial resistance in Taiwan among Gram-positive pathogens: the Taiwan Surveillance of Antimicrobial Resistance (TSAR) programme, 2000. Int J Antimicrob Agents 23: 362–370. 15081085 10.1016/j.ijantimicag.2003.09.021

[22] Naimi TS, LeDell KH, Como-Sabetti K, Borchardt SM, Boxrud DJ, et al (2003) Comparison of community- and health care-associated methicillin-resistant Staphylococcus aureus infection.[see comment]. JAMA 290: 2976–2984. 14665659 10.1001/jama.290.22.2976

[23] Chen ML, Chang SC, Pan HJ, Hsueh PR, Yang LS, et al (1999) Longitudinal analysis of methicillin-resistant Staphylococcus aureus isolates at a teaching hospital in Taiwan. J Formos Med Assoc 98: 426–432. 10443067

[24] Hsueh PR, Liu CY, Luh KT (2002) Current status of antimicrobial resistance in Taiwan. Emerg Infect Dis 8: 132–137. 11897063 10.3201/eid0802.010244 PMC3369580

[25] Chambers HF (2001) The changing epidemiology of Staphylococcus aureus? Emerg Infect Dis 7: 178–182. 11294701 10.3201/eid0702.010204 PMC2631711

[26] Chen CJ, Huang YC, Chen C-J, Huang Y-C (2005) Community-acquired methicillin-resistant Staphylococcus aureus in Taiwan. Journal of Microbiology, Immunology & Infection 38: 376–382. 16341337

[27] Zetola N, Francis JS, Nuermberger EL, Bishai WR (2005) Community-acquired meticillin-resistant Staphylococcus aureus: an emerging threat. Lancet Infect Dis 5: 275–286. 15854883 10.1016/S1473-3099(05)70112-2

[28] Huang YC, Su LH, Wu TL, Lin TY, Huang Y-C, et al (2006) Changing molecular epidemiology of methicillin-resistant Staphylococcus aureus bloodstream isolates from a teaching hospital in Northern Taiwan. Journal of Clinical Microbiology 44: 2268–2270. 16757637 10.1128/JCM.00776-06 PMC1489411

[29] Huang YC, Ho CF, Chen CJ, Su LH, Lin TY (2008) Comparative molecular analysis of community-associated and healthcare-associated methicillin-resistant Staphylococcus aureus isolates from children in northern Taiwan. Clinical Microbiology & Infection 14: 1167–1172. 19076845 10.1111/j.1469-0691.2008.02115.x

[30] Wang JL, Chen SY, Wang JT, Wu GH, Chiang WC, et al (2008) Comparison of both clinical features and mortality risk associated with bacteremia due to community-acquired methicillin-resistant Staphylococcus aureus and methicillin-susceptible S. aureus. Clin Infect Dis 46: 799–806. 18266610 10.1086/527389

[31] Hsiao CH, Chuang CC, Tan HY, Ma DH, Lin KK, et al (2012) Methicillin-Resistant Staphylococcus aureus Ocular Infection: A 10-Year Hospital-Based Study. Ophthalmology 119: 522–527. 22176801 10.1016/j.ophtha.2011.08.038

[32] Cosgrove SE, Sakoulas G, Perencevich EN, Schwaber MJ, Karchmer AW, et al (2003) Comparison of mortality associated with methicillin-resistant and methicillin-susceptible Staphylococcus aureus bacteremia: a meta-analysis. Clin Infect Dis 36: 53–59. 12491202 10.1086/345476

[33] Melzer M, Eykyn SJ, Gransden WR, Chinn S (2003) Is methicillin-resistant Staphylococcus aureus more virulent than methicillin-susceptible S. aureus? A comparative cohort study of British patients with nosocomial infection and bacteremia. Clin Infect Dis 37: 1453–1460. 14614667 10.1086/379321

[34] Skiest DJ, Brown K, Cooper TW, Hoffman-Roberts H, Mussa HR, et al (2007) Prospective comparison of methicillin-susceptible and methicillin-resistant community-associated Staphylococcus aureus infections in hospitalized patients. J Infect 54: 427–434. 17070598 10.1016/j.jinf.2006.09.012

[35] Hawser SP, Bouchillon SK, Hoban DJ, Dowzicky M, Babinchak T (2011) Rising incidence of Staphylococcus aureus with reduced susceptibility to vancomycin and susceptibility to antibiotics: a global analysis 2004–2009. Int J Antimicrob Agents 37: 219–224. 21239146 10.1016/j.ijantimicag.2010.10.029

[36] Fridkin SK, Hageman J, McDougal LK, Mohammed J, Jarvis WR, et al (2003) Epidemiological and microbiological characterization of infections caused by Staphylococcus aureus with reduced susceptibility to vancomycin, United States, 1997–2001. Clin Infect Dis 36: 429–439. 12567300 10.1086/346207

[37] Gordon YJ (2001) Vancomycin prophylaxis and emerging resistance: are ophthalmologists the villains? The heroes? Am J Ophthalmol 131: 371–376. 11239872 10.1016/s0002-9394(00)00955-7

[38] CDC issues recommendations for preventing spread of vancomycin resistance. Am J Health Syst Pharm 52: 1272–1274. 10.1093/ajhp/52.12.1272 7656113

[39] Force A-CT (1999) The prophylactic use of vancomycin for intraocular surgery. Quality of Care Publications, Number 515, American Academy of Ophthalmology, San Francisco, CA, October.

[40] Marangon FB, Miller D, Muallem MS, Romano AC, Alfonso EC (2004) Ciprofloxacin and levofloxacin resistance among methicillin-sensitive Staphylococcus aureus isolates from keratitis and conjunctivitis. American Journal of Ophthalmology 137: 453–458. 15013867 10.1016/j.ajo.2003.10.026

[41] Huang YC, Chen CJ (2011) Community-associated meticillin-resistant Staphylococcus aureus in children in Taiwan, 2000s. Int J Antimicrob Agents 38: 2–8. 21397461 10.1016/j.ijantimicag.2011.01.011

[42] Broekema NM, Van TT, Monson TA, Marshall SA, Warshauer DM (2009) Comparison of cefoxitin and oxacillin disk diffusion methods for detection of mecA-mediated resistance in Staphylococcus aureus in a large-scale study. J Clin Microbiol 47: 217–219. 19020073 10.1128/JCM.01506-08 PMC2620872

[43] Chen FJ, Hiramatsu K, Huang IW, Wang CH, Lauderdale TL (2009) Panton-Valentine leukocidin (PVL)-positive methicillin-susceptible and resistant Staphylococcus aureus in Taiwan: identification of oxacillin-susceptible mecA-positive methicillin-resistant S. aureus. Diagnostic Microbiology and Infectious Disease 65: 351–357. 19766426 10.1016/j.diagmicrobio.2009.07.024

